# Drilling Damage and Dynamic Response of Carbon/Glass Hybrid Nomex Sandwich Composites

**DOI:** 10.3390/polym18182209

**Published:** 2026-09-10

**Authors:** Ibrahim Demirci

**Affiliations:** Department of Mechatronics Engineering, Technology Faculty, Selcuk University, 42075 Konya, Turkey; ibrahim.demirci@selcuk.edu.tr

**Keywords:** Nomex honeycomb, hybrid composites, drilling-induced damage, dynamic analysis, image processing

## Abstract

Nomex honeycomb core sandwich composites are attractive for lightweight structural applications because of their high specific stiffness. However, drilling required for mechanical joining can introduce both surface and subsurface damage and may change the mechanical and dynamic response of the structure. This study examines Nomex aramid honeycomb sandwich composites with C/C/C, C/G/C, G/C/G, and G/G/G face-sheet configurations using three-point bending, drilling, image analysis, ultrasonic C-scan, and acoustic impact measurements. The C/C/C configuration reached an average bending load 36.05% higher than G/G/G and reduced the entry and exit side delamination areas by 88.91% and 78.89%, respectively. Although C/C/C produced the highest thrust forces during drilling, it also showed the smallest surface and internal damage regions, indicating that thrust force alone does not fully describe hole quality. C/G/C also exhibited less damage than G/C/G; however, these two hybrid configurations differ in both carbon/glass content and ply position, so the contribution of each factor cannot be separated independently. After drilling, the natural frequency decreased, and the damping ratio increased in all configurations. Overall, the carbon-rich face sheets provided higher bending resistance and more effectively limited drilling-induced damage.

## 1. Introduction

Sandwich composite structures are multilayered structures created by combining two thin, high-strength surface layers with a low-density and relatively thick core material. Their ability to provide high flexural and torsional strengths despite their low weight allows for their widespread use in engineering fields, such as aerospace and automotive [1,2,3,4,5]. Sandwich panels, especially those with fiber-reinforced polymer surface layers, are also being considered in load-bearing panel applications where high structural performance combined with low weight is required [5]. The majority of tensile and compressive stresses generated under bending loads are carried by the surface layers. The core material contributes to bending rigidity by maintaining the distance between the surfaces and undertakes the transmission of shear loads [1,6]. This structural arrangement allows for the achievement of high specific stiffness, specific strength, and energy absorption capacity along with low weight. These properties play a significant role in the preference for sandwich composites in applications requiring advanced technology [7,8].

Honeycomb structures are among the commonly used core types in sandwich composites. Aramid-based honeycomb cores offer significant advantages in lightweight structural applications due to their low density, high out-of-plane compressive and shear performance, fatigue resistance, and energy absorption capacity [9]. Combining aramid honeycomb cores with carbon or glass fiber-reinforced polymer (CFRP/GFRP) surface layers enables the production of structures with high flexural rigidity at low mass. The cellular and anisotropic structures of the honeycomb core prevent load transfer and damage development from occurring homogeneously throughout the structure. The mechanical and dynamic behaviors are affected by the properties of the core and the material type, thickness, and laminated arrangement of the surface layers [10,11,12,13,14].

A significant portion of research on the processability of structural composites has focused on the perforation of monolithic fiber-reinforced polymer laminates [15]. Research on aramid honeycomb core sandwich panels has been relatively limited [16]. Deformation of the cellular and anisotropic core, along with the surface layers, can lead to the simultaneous development of different damage mechanisms during drilling [15]. Geier and Díaz-Álvarez et al. reported that systematic experimental studies on drilling-induced damage in aramid-based sandwich composites remain limited. In recent years, studies on the drillability of CFRP/GFRP face-sheet sandwich structures under different drill geometries, cutting speeds, and feed rates have increased. At this point, it is expected that the damage caused during drilling will be affected not only by the structure of the honeycomb core but also by the material type and layer arrangement of the surface layers [15,17].

Carbon fiber reinforced epoxy composites stand out in structural applications due to their high specific strength, high specific elastic modulus, and good fatigue behavior [18,19]. Glass fiber reinforced polymer composites, on the other hand, have different advantages such as lower cost, higher fracture toughness, wide deformation capacity, and relatively good impact resistance [19,20,21]. The hybrid use of carbon and glass fiber in the same structure allows for the combined utilization of the different mechanical properties of the two reinforcement systems and reduces the cost associated with carbon fiber use [18,19,20,21,22].

The stiffness, damage tolerance, fracture behavior, vibration response, and cost of hybrid composites can vary depending on their layer arrangement [23,24]. The position of the carbon and glass fibers along the thickness direction also affects the behavior of the structure [23,24]. In loading conditions where surface layers are effective, such as bending and vibration, the presence of high-stiffness carbon layers in the outer or inner regions can significantly alter the mechanical response [23,24]. Previous studies have also reported that the unconfined and forced vibration behaviors of carbon/glass hybrid layers are affected by the layer arrangement and boundary conditions [23,24,25,26,27].

The carbon/glass surface layer arrangement affects the mechanical and dynamic behavior of the sandwich structure, and the drilling process applied during the mechanical joining of these structures can cause additional damage to the surface layers and the core [28,29,30,31]. The heterogeneous and anisotropic nature of composites renders their drilling behavior more complex than that of traditional metallic materials [28,29,30,31]. When an axial force is applied by the cutting tool, various damage mechanisms can develop, such as matrix cracking, fiber breakage, fiber pulling, surface layer-core separation, delamination, burr formation, and crushing of honeycomb cell walls. These damages can impair the hole geometry and connection quality, as well as reduce the load-bearing capacity and service life of the structure [28,29,30,31,32,33,34,35].

The thrust force measured during drilling is a fundamental parameter used to evaluate the machinability and damage around the hole [36,37,38,39]. If the thrust force exceeds the interlayer strength of the surface layers, it can lead to peeling at the drill entry and thrust-type delamination at the exit surface. The magnitude of the force is affected by the feed rate, rotational speed, tool geometry, tool wear, surface layer material, layer arrangement, and core structure [29,36,37,38,39]. In aramid honeycomb core sandwich composites, it has been reported that, in addition to thrust force and torque, burrs and surface defects related to the cell structure also affect hole quality. Studies on glass fiber/epoxy composites have also investigated the effects of drilling parameters on thrust force and delamination [36,37,38,39].

However, identifying geometric damage around the hole alone is not sufficient to explain the overall behavior of the structure after drilling. Delamination, surface layer/core separation, and matrix cracks can also affect the dynamic response by altering structural rigidity and energy loss mechanisms [40,41].

These criteria allow for the comparison of visible damage around the hole but do not directly reflect the change in the dynamic behavior of the structure caused by the damage [42]. Delamination, surface-core separation, and matrix cracks can alter the mass, stiffness, and damping properties of structures. Local reductions in stiffness can lead to changes in natural frequencies, increases in vibration amplitudes, displacement of resonance peaks, and differentiation of energy distribution. Previous studies have reported that delamination zones can generate local bending resonances and can be identified using vibration-based approaches. Investigating vibrational and acoustic responses after drilling allows for the evaluation of information obtained from geometric damage criteria concerning the dynamic behavior of the structure [40,41,42,43,44,45,46,47].

Vibration and acoustic measurements can be used to investigate stiffness loss and damage development in composite structures non-destructively [48,49,50,51]. The sound pressure level, dominant frequencies, spectral energy, and resonance-related changes can be determined from the acoustic pressure signals. It has been previously reported that the vibroacoustic behavior of honeycomb-core sandwich panels is affected by the core geometry and surface properties [10,52]. There are studies in the literature on the evaluation of vibration and acoustic behavior of sandwich composites and drilling-induced damage [9,42,47,48,49,50,51]. However, these studies mostly address drilling damage, acoustic response, or vibration behavior separately. Studies that evaluate the effects of carbon/glass hybrid surface layer arrangement on thrust force generated during drilling, surface delamination, internal damage, and post-drilling dynamic properties together in the same experimental study are limited [10,47,48,49,50,51,52].

Previous studies have generally examined the mechanical behavior of carbon/glass hybrid face sheets, drilling-induced damage, and the dynamic response of sandwich structures as separate topics. However, studies that consider these effects together for Nomex honeycomb core hybrid sandwich composites remain limited. In this study, Nomex aramid honeycomb core sandwich composites with C/C/C, C/G/C, G/C/G, and G/G/G face-sheet configurations were investigated. The effect of the stacking sequence on the flexural behavior and drilling performance was evaluated through three-point bending tests and thrust force measurements. Surface damage after drilling was quantified by image processing, and internal damage was examined using ultrasonic C-scan. In addition, acoustic impact measurements were performed before and after drilling to determine the changes in the natural frequency and damping ratio. Thus, the effects of different face-sheet arrangements on the mechanical performance, drilling damage, and dynamic behavior were evaluated.

## 2. Materials and Methods

### 2.1. Materials and Sandwich Composite Production

In this study, sandwich composites with a Nomex aramid honeycomb core and carbon/glass fiber-reinforced surface layers were produced. The Nomex honeycomb used as the core material had a cell width of 3.2 mm, thickness of 10 mm, and density of 48 kg/m^3^. The top and bottom surfaces of the sandwich composites were composed of three layers. Therefore, six fiber fabric layers were used in each panel. In the production of the surface layers, carbon and glass fiber fabrics with area densities of 400 g/m^2^ and 390 g/m^2,^ respectively, and a twill weave structure were used. The Hexion MGS L285/H287 epoxy resin system (Hexion Specialty Chemicals, Stuttgart, Germany) was selected as the matrix material.

**Figure 1:**
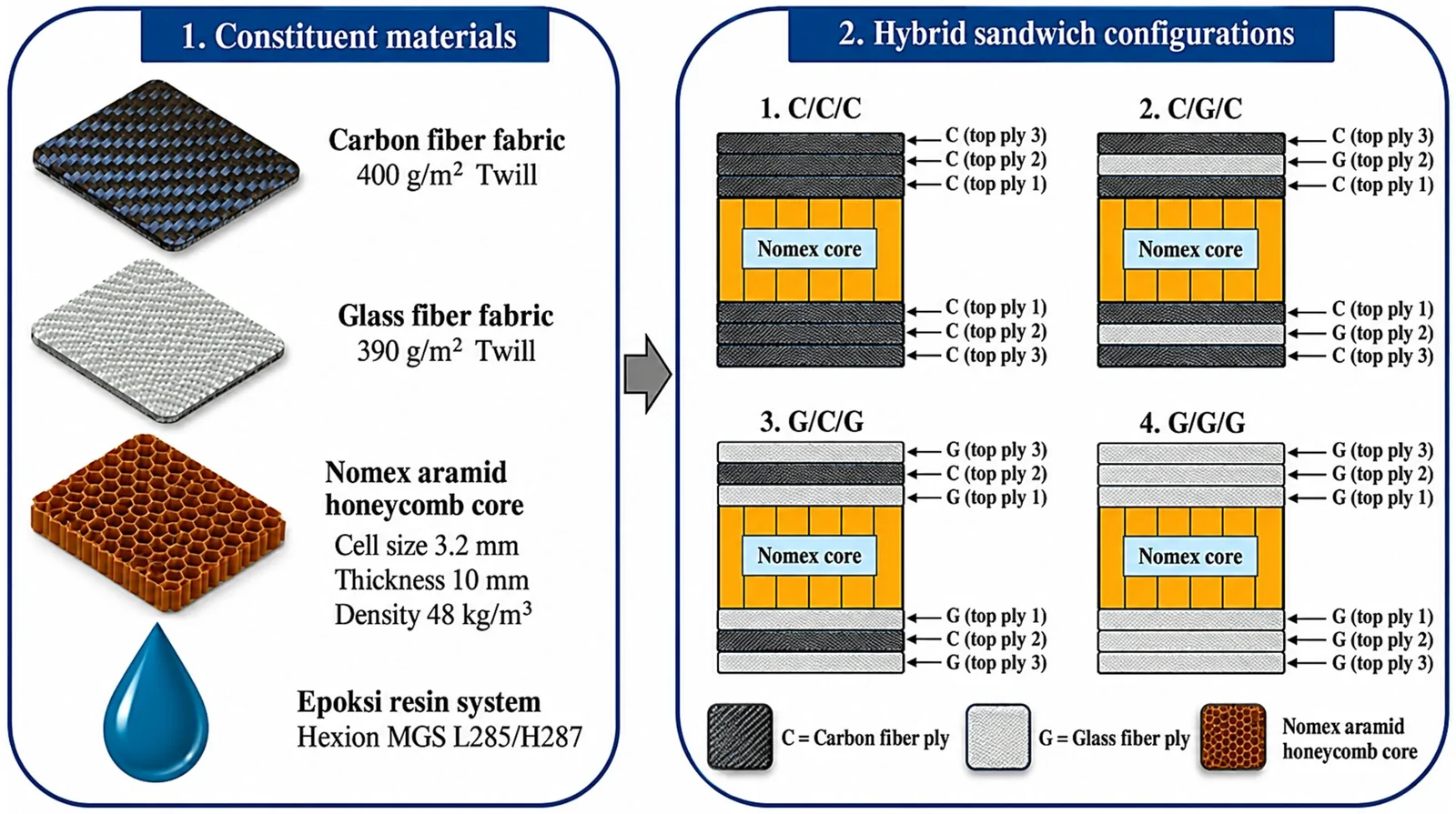
Hybridization scheme of C/C/C, C/G/C, G/C/G and G/G/G sandwich composites.

Four surface layer configurations were chosen to compare the combined effect of carbon/glass composition and layer arrangement on structural behavior. Since C/G/C and G/C/G configurations differ in both carbon/glass ratio and layer position, the independent contributions of these two effects could not be separated with the current experimental setup. The C/C/C and G/G/G configurations were used as reference groups representing fully carbon- and fully glass-fiber face sheets, respectively. The C/G/C and G/C/G configurations represent two hybrid arrangements in which carbon and glass fiber layers occupy different positions within the three-ply face sheet. This arrangement allowed the mechanical behavior, drilling-induced damage, and dynamic response of the all-carbon, all-glass, carbon-rich, and glass-rich configurations to be compared under the same test conditions. All configurations were applied symmetrically to the upper and lower sides of the Nomex honeycomb core to minimize structural differences other than the face-sheet arrangement. The hybridization scheme of the sandwich composites is illustrated in Figure 1.

The sandwich composite panels were produced using a hand lay-up method. Before fabrication, the carbon and glass fiber fabrics and Nomex honeycomb core were cut to the same panel dimensions. Hexion MGS L285 epoxy resin and H287 hardener were mixed at a weight ratio of 100:40 and stirred until a uniform mixture was achieved. The measured fiber volume fraction of the face sheets was approximately 45–50% for all the configurations. The three plies forming the lower face sheet were arranged according to the C/C/C, C/G/C, G/C/G, and G/G/G stacking sequences. The resin mixture was applied manually to each ply until the fabric was fully wetted. The Nomex honeycomb core was then placed on the impregnated lower face sheet of the panel. The upper face sheet was prepared similarly and positioned symmetrically on the opposite side of the core. Bonding between the face sheets and the Nomex core was achieved using the same epoxy system during curing.

**Figure 2:**
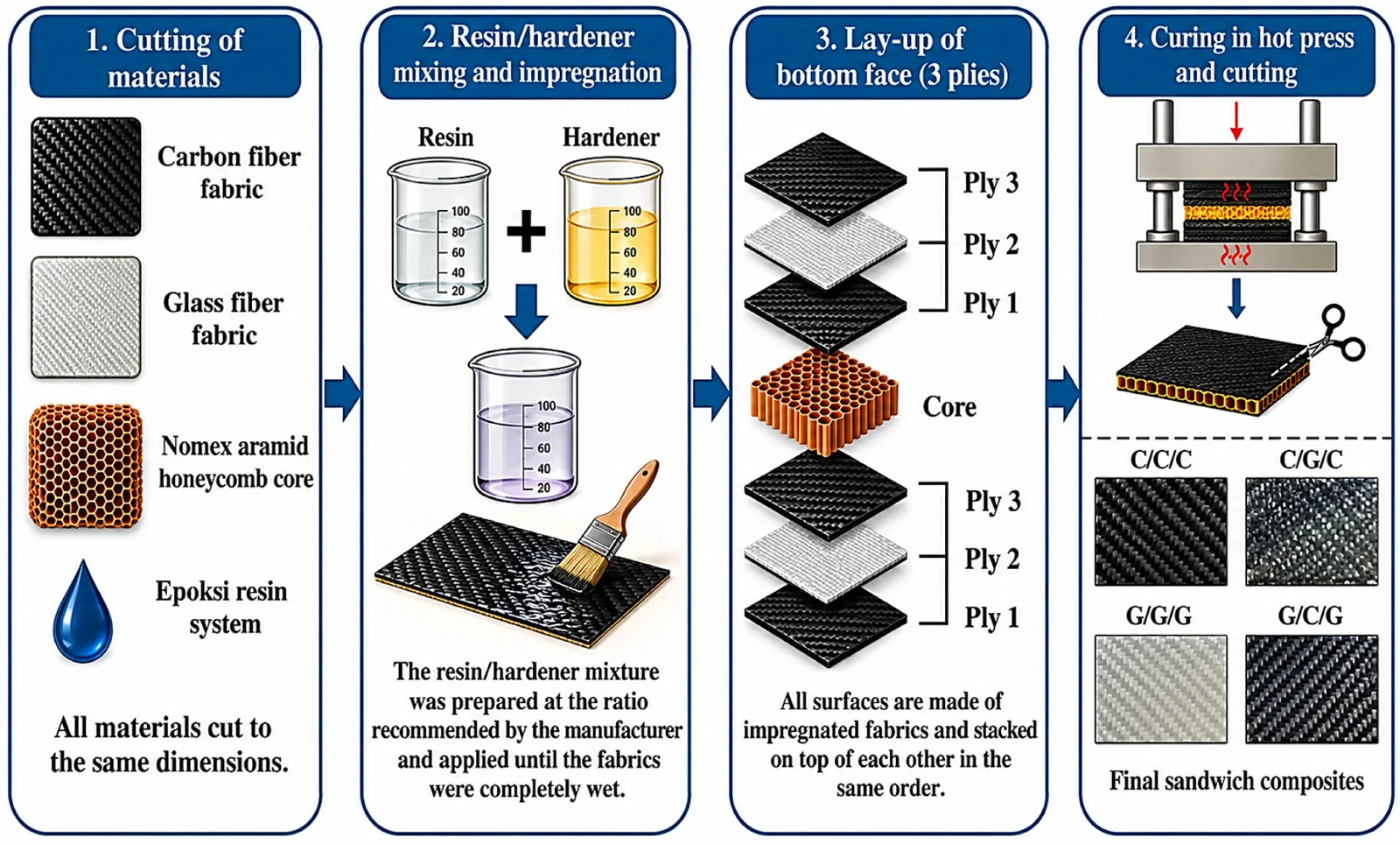
Production scheme of C/C/C, C/G/C, G/C/G and G/G/G sandwich composites.

The assembled panels were cured in a hot press at 120 °C and 100 bar for 5 h. Excess epoxy was expelled during pressing to limit the resin accumulation within the face sheets. After curing, the thickness of each three-ply face sheet was approximately 2 mm each. Together with the 10 mm Nomex honeycomb core, the final panel thickness was approximately 14 mm. The final masses of the panels produced with the same dimensions ranged from 106.352 to 106.714 g. The same resin/hardener ratio, number of plies, fabrication procedure, pressing pressure, curing temperature, and curing time were used for all the specimen groups. Only the stacking sequence of the carbon and glass fiber plies was varied for the specimens. After curing, the panels were removed from the mold and cut to the required dimensions for the three-point bending and the drilling tests. The production scheme of C/C/C, C/G/C, G/C/G, and G/G/G sandwich composites is shown in Figure 2.

### 2.2. Three-Point Bending Experiment

Three-point bending tests were performed to determine the flexural and core shear behavior of the sandwich composites according to ASTM C393/C393M-16 [53]. The tests were carried out on a Shimadzu AGS-X 100 kN universal testing machine (Shimadzu Corporation, Kyoto, Japan) at a loading rate of 5 mm/min. Specimens were prepared with dimensions of 130 × 40 mm, and a support span of 70 mm was selected to suit the specimen geometry used in this study. The tests were performed three times for each group of specimens.

Load and displacement data were continuously recorded throughout the experiment. In addition to the maximum load, the core shear stress (*F_s_*) and surface layer stress (σ_f_) at maximum load were calculated using the relationships based on ASTM C393/C393M-16:

$$F_{s}\;=\;\frac{Pmax}{(d+c)b}\;\sigma_{f}\;=\;\frac{PmaxS}{2t(d+c)b}$$

Here, *P*_max_ represents the maximum load, *d* the total sandwich thickness, *c* the core thickness, *b* the sample width, *t* the thickness of a surface layer, and *S* the support span. The values used in the calculations were 14, 10, 40, 2, and 70 mm, respectively.

**Figure 3:**
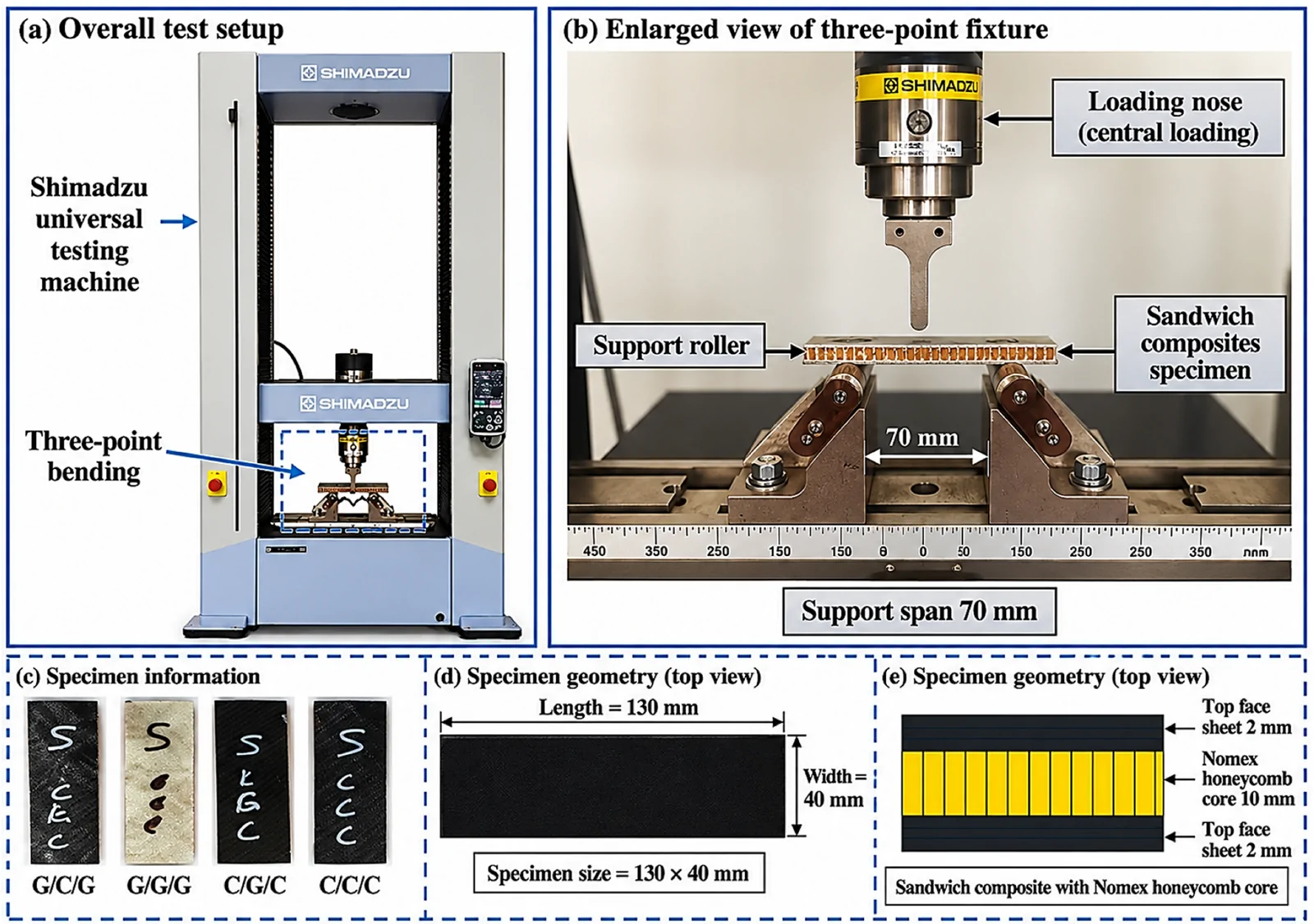
Three-point bending test setup of Nomex aramid honeycomb core sandwich composite specimens: (**a**) General view of the Shimadzu AGS-X 100 kN universal testing machine, (**b**) three-point bending fixture with a 70 mm support span, and (**c**) schematic representation of the sandwich structure with test specimens measuring 130 × 40 mm.

The three-point bending test setup for the C/C/C, C/G/C, G/C/G, and G/G/G sandwich composites is shown in Figure 3.

### 2.3. Drilling Process

Drilling tests were performed on C/C/C, C/G/C, G/C/G, and G/G/G sandwich composite specimens using a CNC-controlled vertical machining center (Linermak, Istanbul, Türkiye). Three specimens were drilled for each face sheet configuration, resulting in a total of 12 drilling tests. The specimens were fixed in a dedicated clamping fixture to limit unwanted movements and vibrations during drilling. The fixture was mounted on a dynamometer to record the axial thrust force throughout the drilling process.

A 7 mm diameter Ti-coated HSS N-type twist drill was used for all tests. The drill had a point and helix angles of 118° and 30°, respectively. To minimize the effect of tool wear on the thrust force and delamination, a new drill with the same geometry was used for each specimen. Therefore, no drill was used for more than one test, which limited the influence of progressive tool wear associated with the drilling sequence. The same clamping procedure and drilling conditions were used for all the specimens.

A relatively low feed was selected to reduce the axial loading and the tendency for delamination in the face sheets. Drilling was performed at 1750 rpm and a feed rate of 50 mm/min, corresponding to approximately 0.029 mm/rev. Previous studies have shown that feed rate has a marked influence on thrust force and delamination, and that lower feed values can help limit these effects [36]. Since the aim of the present study was not to optimize the drilling parameters, but to compare the drilling response of different carbon/glass face-sheet configurations, the spindle speed and feed rate were kept constant for all tests.

**Figure 4:**
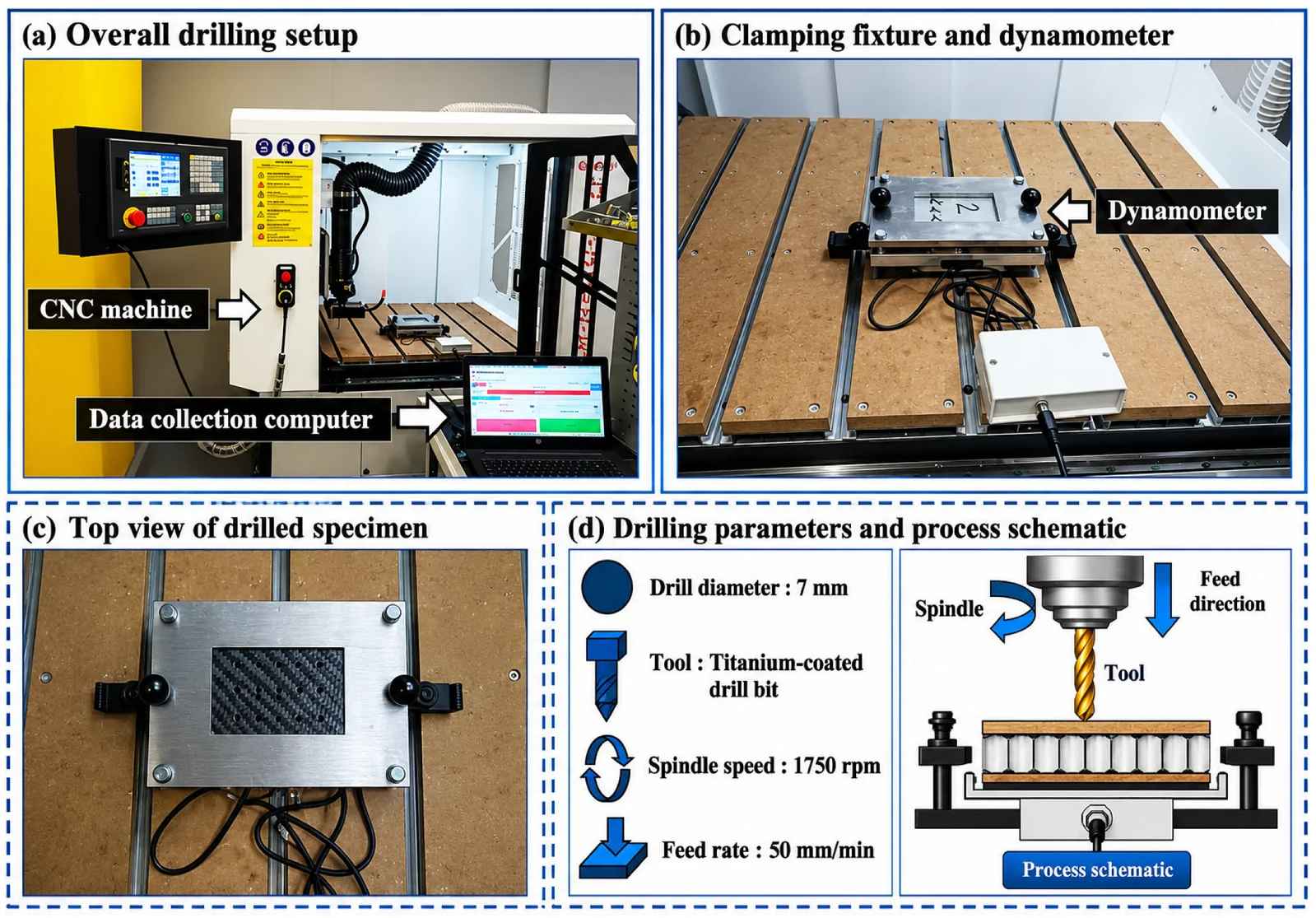
Drilling test setup and measurement system used: (**a**) general view of the CNC-controlled drilling system, (**b**) dynamometer and clamping apparatus, (**c**) top view of the drilled specimen, and (**d**) schematic representation of the drilling process with applied drilling parameters (drill diameter: 7 mm, spindle speed: 1750 rpm, feed rate: 50 mm/min).

During drilling, the thrust force along the drill axis was recorded continuously using the dynamometer (Yordam Test Ölçüm ve Mühendislik, Istanbul, Türkiye). Peak thrust-force values were obtained from the force–time data and used to compare the different face-sheet configurations. After drilling, the damage around the hole on both the entry and exit surfaces was examined to assess the delamination behavior. The drilling experimental setup is illustrated in Figure 4.

### 2.4. Delamination Characterization and Image Processing

**Figure 5:**
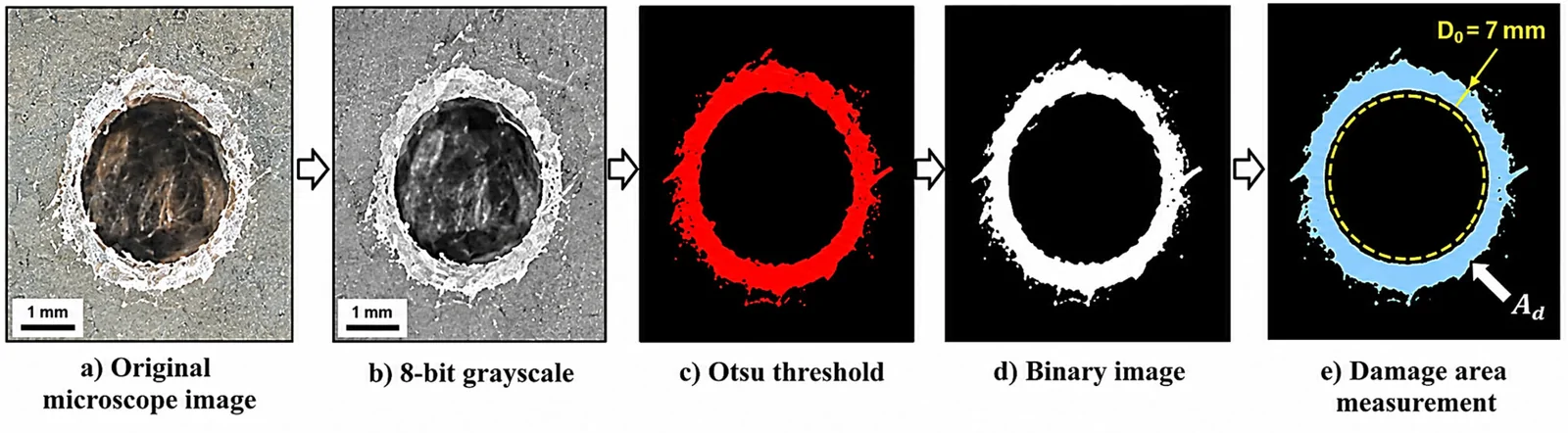
Image-processing workflow for quantifying drilling-induced delamination damage. Red regions indicate the thresholded damage area, white regions represent the binary damage mask, the light-blue region represents the measured damage area, and the yellow dashed circle indicates the nominal 7 mm hole boundary.

The images were first converted to 8-bit grayscale in ImageJ (Version 2, National Institutes of Health, Bethesda, MD, USA). Spatial calibration was performed using the nominal hole diameter of 7 mm with the Set Scale function, allowing the measured areas to be converted from pixels to mm^2^. Damage boundaries were identified using Otsu’s automatic thresholding method, and the thresholded images were converted to binary form. The Fill Holes function was then applied to obtain continuous damage regions. Isolated regions and image features unrelated to drilling damage were removed manually. The nominal hole area was excluded from the measurement, and only the damaged area extending beyond the original hole boundary was considered. The same calibration, thresholding, filling, and cleaning procedure was applied to all entry and exit images. The main steps of this procedure are shown in Figure 5.

An additional threshold sensitivity analysis was performed to evaluate the segmentation uncertainty associated with Otsu thresholding. For each image, the threshold value determined by the Otsu method was taken as the reference value (*T*), and the damage area was recalculated at threshold levels 0.90*T* 0.95*T*, *T*, 1.05*T*, and 1.10*T*. During this analysis, all image processing steps, except for the threshold value, were applied without change. The damaged area obtained at each threshold level was compared with the reference Otsu result to determine the percentage change in the damaged area. Current studies indicate that area-based measurements are advantageous, especially when the damage pattern is irregular [54].

After removing small and isolated image noises, white pixel regions outside the nominal hole boundary were considered as delamination areas. The same image processing parameters were applied to all C/C/C, C/G/C, G/C/G, and G/G/G sandwich samples.

Drilling-induced damage was quantified using the area-based delamination factor ($F_{d}$) and the equivalent delamination factor ($F_{eq}$). The nominal hole area was calculated as

A_0_ = πD_0_^2^/4

where $D_{0}$ is the nominal hole diameter, taken as 7 mm in this study. The damage area identified outside the nominal hole boundary by image processing was denoted as $A_{d}$, and the total affected area was calculated as

*A_t_* = *A*_0_ + *A_d_*

The area-based delamination factor was then determined from

*F_d_* = 1 + (*A_d_*/*A*_0_)

and the equivalent delamination factor was obtained from

$$D_{\mathrm{eq}}=\sqrt{4At/\;\pi }$$

*F_eq_ = D_eq_/D*_0_

Accordingly, $F_{d}$ represents the ratio of the total affected area to the nominal hole area, whereas $F_{eq}$ expresses the same damage in terms of a normalized equivalent diameter.

Entry and exit surfaces were evaluated separately, and the results were reported as mean values, standard deviations, and coefficients of variation (CoV).

### 2.5. Acoustic Impact and Dynamic Response Analysis

The dynamic response of the specimens before and after drilling was investigated using an acoustic pulse method. Drilled and undrilled specimens were tested under the same boundary conditions, and a controlled impact force of 5 N was applied to each specimen using an impact hammer. The same specimens were measured before and after drilling so that the change in dynamic response could be evaluated without introducing specimen-to-specimen variability. All measurements were performed under identical boundary conditions. The resulting acoustic response was recorded with a microphone positioned close to the impact region. For each specimen, three measurements were performed at positions spaced 120° apart around the drilled hole, and the corresponding spectra were averaged.

The acoustic signals were sampled at 22,050 Hz and transformed from the time domain to the frequency domain using a 2048-point Fast Fourier Transform (FFT). A Hann window was applied to reduce spectral leakage. These settings provided a frequency resolution of approximately 10.77 Hz. The natural frequencies were estimated from the dominant resonance peaks in the acoustic FFT spectra. Since the time history of the applied impact force was not recorded separately, a conventional force–response frequency response function (FRF) was not calculated. Therefore, the reported natural frequencies represent resonance frequencies identified from the measured acoustic response.

**Figure 6:**
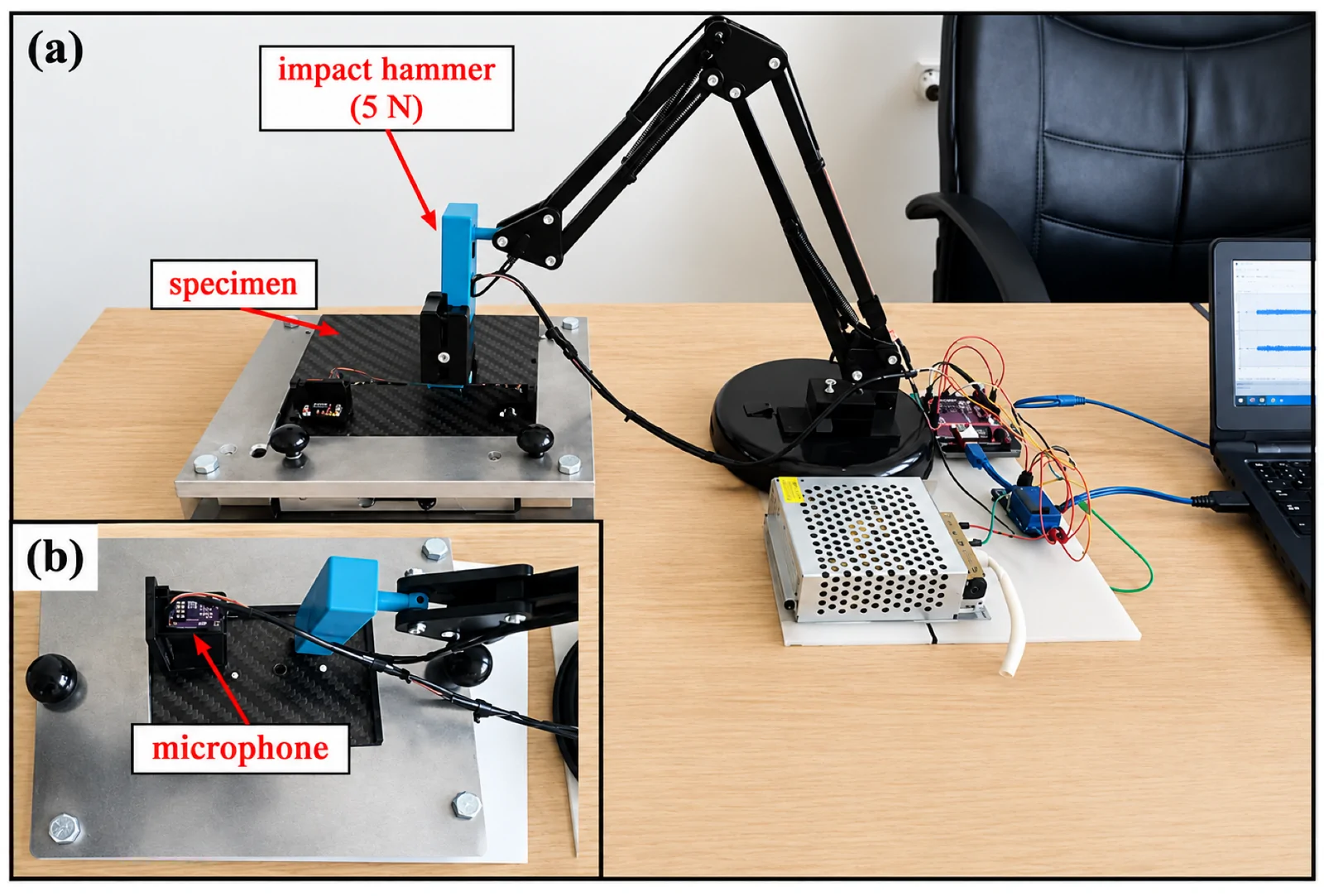
Impulse-induced vibration and acoustic measurement setup: (**a**) general view, (**b**) microphone position.

The damping ratio was determined from the selected resonance peak using the half-power bandwidth method. For each peak, $f_{n}$ denotes the resonance frequency, while $f_{1}$ and $f_{2}$ are the lower and upper frequencies at which the spectral amplitude decreases to $1/\sqrt{2\;}$(approximately 0.707) of the peak amplitude. The damping ratio was calculated as:

$$\zeta \;=\frac{f2-f1}{2fn}$$

where ζ is the damping ratio. The same signal-processing procedure was applied to all specimens to compare the changes in natural frequency and damping ratio associated with drilling-induced damage. The impact-induced acoustic measurement setup is shown in Figure 6.

### 2.6. Ultrasonic C-Scan Analysis

Ultrasonic C-scan measurements were performed using a computer-controlled immersion scanning system to examine internal damage produced by drilling (CRD Endüstriyel Kontrol Sistemleri, Bursa, Türkiye). The specimens were fixed in a water tank, with water serving as the acoustic coupling medium. The distance between the probes was set to 54 mm. Based on the selected X–Y start and end coordinates, the scanned area was approximately 116 × 129 mm, and the spatial positioning resolution of the system was 1 mm.

Data were acquired at a sampling frequency of 100 MHz in RF mode. The acquisition window was defined using a 12 µs delay and a 50 µs depth setting. Averaging was used during signal acquisition, with the amplitude level set to Level 16 and the pulse duration to 3 µs. A 1–6 MHz band-pass filter was selected. The preamplifier was set to 0 dB, while the gain was fixed at 55 dB in CONST mode. The trigger source was INT, and the trigger frequency was set to 4 kHz. The same acquisition and scanning settings were used for all specimen groups.

Internal drilling damage was identified as continuous low-amplitude regions surrounding the hole that were clearly distinguishable from the intact material. Periodic and isolated indications corresponding to the 3.2 mm cell geometry of the Nomex honeycomb core were excluded from the damage assessment. The same damage criterion was applied to the C/C/C, C/G/C, G/C/G, and G/G/G configurations.

**Figure 7:**
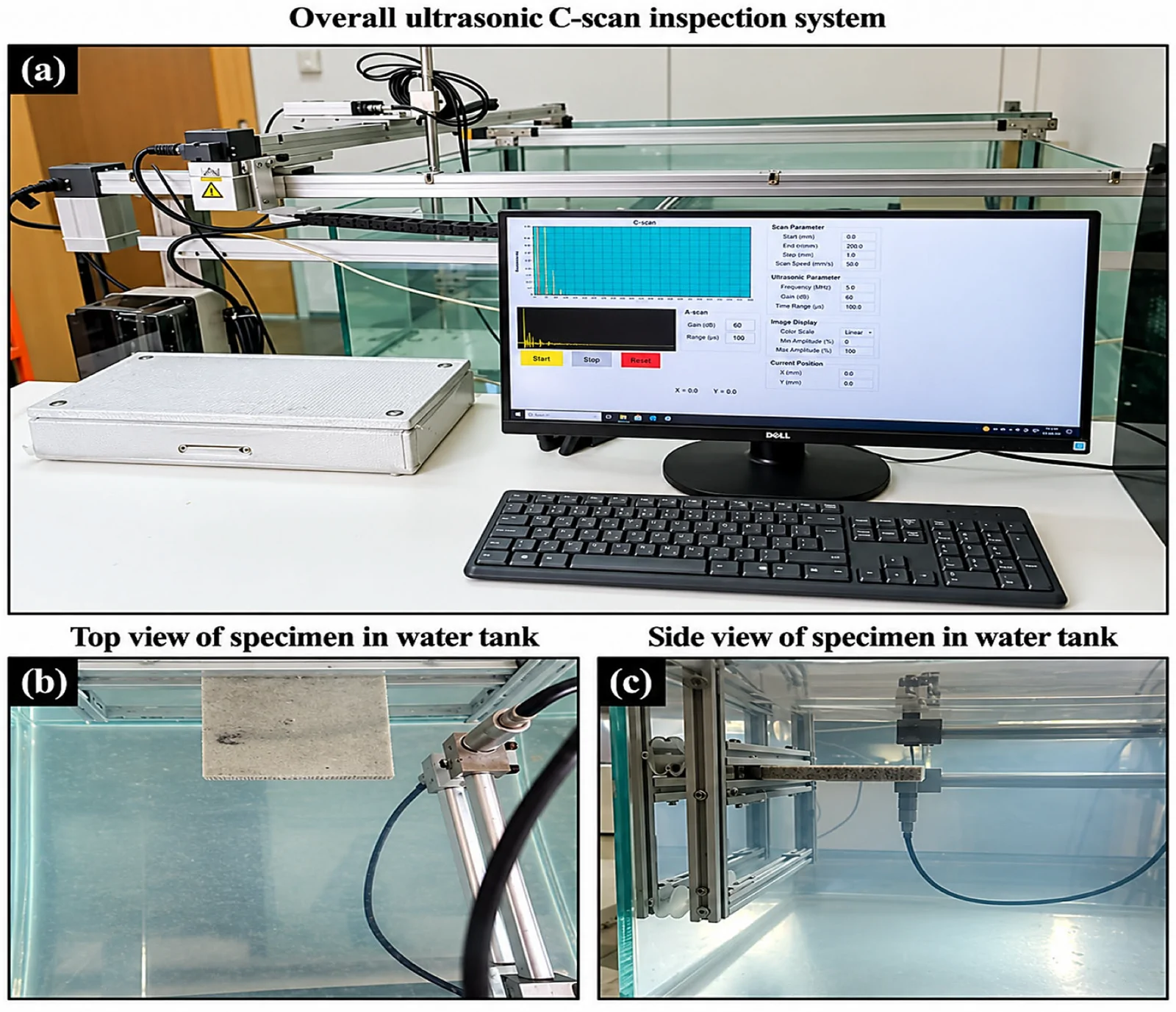
Ultrasonic C-scan analysis system: (**a**) general view, (**b**) top view of the sample in the water tank, and (**c**) side view.

For quantitative evaluation, the continuous low-amplitude regions outside the nominal hole boundary were segmented from the C-scan maps and their total internal damage area was calculated. Three repeated scans were evaluated for each configuration, and the results were reported as mean internal damage area, standard deviation, and coefficient of variation (CoV). This allowed the internal damage obtained from C-scan measurements to be compared directly with the entry- and exit-surface delamination areas determined from optical image processing. The ultrasonic C-scan analysis system is shown in Figure 7.

## 3. Experimental Results

### 3.1. Three-Point Bending Test and Microscopic Damage Analysis

The damage mechanisms of Nomex honeycomb core sandwich composites with C/C/C, C/G/C, G/C/G, and G/G/G surface layer arrangements were investigated after three-point bending using microscopic images and load–displacement curves. During bending, predominantly compressive stress occurred in the upper surface layer, tensile stress in the lower surface layer, and shear and local compressive stress in the core. After the experiment, different types of damage, such as surface layer delamination, fiber fracture, surface wrinkling, core cell wall buckling, and core rupture, were determined. It was evaluated that these damages developed interactively with each other during the bending process.

**Figure 8:**
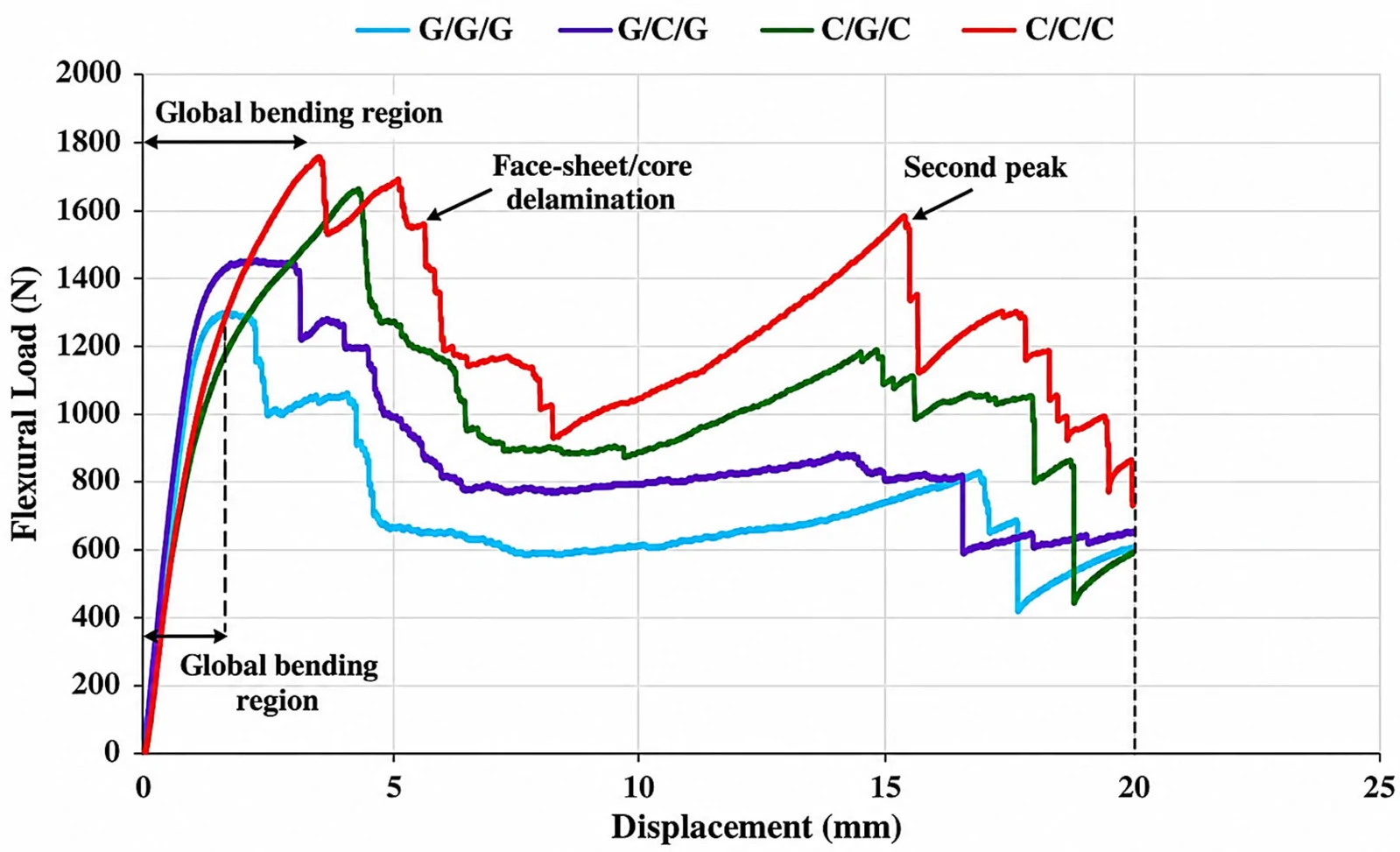
Three-point bending behavior of different surface layer configurations: load–displacement curves and damage development regions. The vertical dashed lines indicate the approximate boundaries of the identified deformation/damage stages.

When the load–displacement curves in Figure 8 are examined, the face-sheet configuration has a clear effect on the flexural response. The highest load-carrying capacity was obtained for the C/C/C configuration, followed by C/G/C, G/C/G, and G/G/G configurations. The sudden drops observed after the peak load were considered to be associated with the development of face-sheet damage, face-sheet/core interfacial debonding, and local damage in the Nomex honeycomb cells observed after testing. The repeated load drops and recoveries after the peak may be associated with progressive damage and the continued load-carrying response of the remaining intact regions and compressed core cells. The average peak load, standard deviation, CoV, core shear stress at maximum load, surface layer stress, and load variations according to G/G/G for the configurations are given in Table 1. The calculated stresses were of the same order as the load results. The C/C/C configuration yielded the highest values, with a core shear stress of 1.80 MPa and a surface layer stress of 31.43 MPa at the maximum load, whereas for G/G/G, these values were determined as 1.32 and 23.11 MPa, respectively.

**Figure 9:**
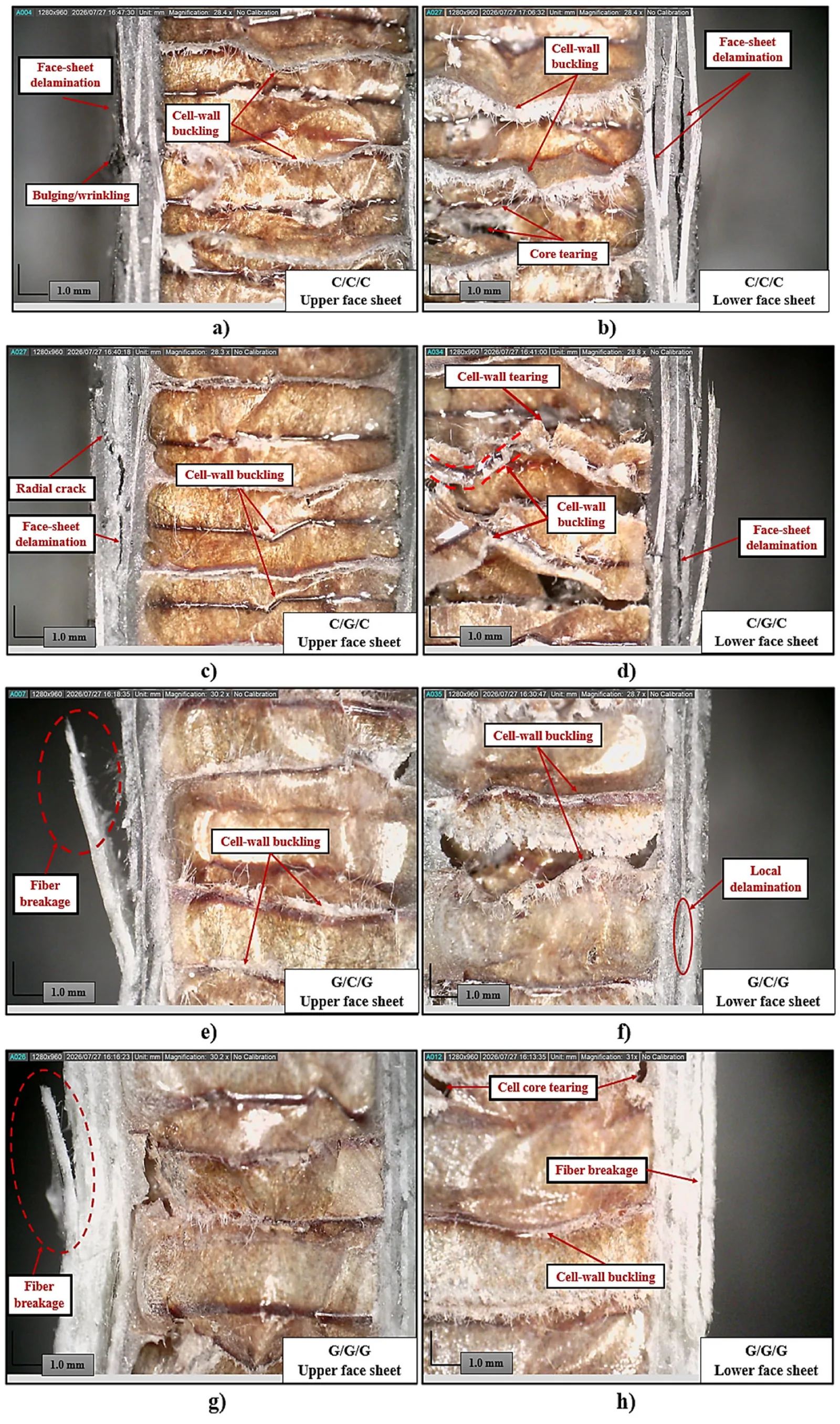
Microscopic images of damage after the three-point bending test: (**a**) C/C/C upper face sheet; (**b**) C/C/C lower face sheet; (**c**) C/G/C upper face sheet; (**d**) C/G/C lower face sheet; (**e**) G/C/G upper face sheet; (**f**) G/C/G lower face sheet; (**g**) G/G/G upper face sheet; and (**h**) G/G/G lower face sheet.

The higher load-carrying capacity of the C/C/C specimens was attributed to the high stiffness of the carbon fiber face sheets. Because of their relatively limited deformation capacity, the damage in these specimens developed more abruptly. Surface wrinkling and local outward deformation were observed on the upper face sheet under compression, along with face-sheet damage and buckling of the Nomex honeycomb cell walls. On the lower side, core-cell buckling and local core rupture were accompanied by damage at the face sheet/core interface. These observations indicate that the compressive instability of the upper face sheet and tensile-side damage in the lower region jointly contributed to the failure process. The second load peak, observed at a displacement of approximately 15 mm, may indicate that the structure retained part of its load-carrying capacity after the initial damage. The subsequent increase in load may be related to the continued contribution of the remaining intact face-sheet regions and compressed core; however, this mechanism was not measured directly in the present study. Microscopic images of the damage occurring after the three-point bending test of the C/C/C, C/G/C, G/C/G, and G/G/G sandwich composites are shown in Figure 9.

Damage observations revealed that a complex damage behavior developed in all configurations rather than a single neat core shear fracture. Surface layer/core interface separation and local core cell buckling were more prominent in the C/C/C and C/G/C samples, whereas surface layer fiber fracture was observed along with core cell buckling in the G/C/G and G/G/G samples. Local core fractures were also observed in the G/G/G samples. Therefore, the dominant damage mode was considered to be a complex damage mode in which surface layer damage, interface separation, and local core cell damage developed simultaneously.

The relatively high load-carrying capacity of the C/G/C specimens is consistent with the higher carbon content and the presence of carbon plies at the outer positions. The stiffness mismatch between the carbon and glass layers may have increased local interlaminar stresses at the hybrid interfaces [55]. Microscopic examination showed radial cracks, local delamination within the face sheet, and buckling of the honeycomb cell walls on the upper side. On the lower side, core rupture, cell-wall buckling, and local interlaminar separation were observed. These damage features suggest that both the more compliant glass ply and the stiffness mismatch at the carbon/glass interfaces affected the failure process. The stepwise damage development observed in the specimens was also consistent with the abrupt load drops in the load–displacement curve.

In the G/C/G specimens, the outer glass plies resulted in a more compliant response, and the damage was distributed over a wider region. Fiber fracture and local buckling of the honeycomb cell walls were observed on the upper side. On the lower side, the damage was less severe and mainly consisted of local delamination within the face sheet and cell-wall buckling. Compared with the C/C/C and C/G/C specimens, the greater deformability of the outer glass plies appears to have spread the deformation over a broader area, reducing the tendency for extensive lower-side damage such as core rupture. At the same time, the lower load-carrying capacity of the G/C/G specimens is consistent with the greater compliance associated with the higher glass-fiber content.

The G/G/G specimens showed the lowest load-carrying capacity among the four configurations, and the damage developed more gradually. Pronounced fiber fracture was observed on the upper face sheet. On the lower side, fiber damage was accompanied by buckling and local rupture of the Nomex honeycomb cells. The microscopic observations indicate that the failure was not confined to the face sheet but also involved the core region. The greater deformability of the glass-fiber plies may have contributed to the more gradual damage development observed in the G/G/G specimens.

The trends observed in the bending experiments were similar to those in previous studies on carbon/glass hybrid composites. Wu et al. reported that the layer arrangement affects the bending behavior, while Han et al. reported that increasing the carbon fiber content improves the bending capacity of honeycomb sandwich structures. These findings are consistent with the higher maximum bending loads observed in the C/C/C and C/G/C configurations in the present study [12,24].

Taken together, the bending results show that the load-carrying capacity increased with increasing carbon-fiber content, particularly when carbon plies were located on the outer surfaces. Damage developed more abruptly in the carbon-rich configurations, whereas the greater deformability of the glass-fiber plies resulted in a more gradual damage progression. In the hybrid specimens, the observed response was associated with the combined effects of carbon/glass composition and ply position. Differences in ply stiffness may also have contributed to the observed damage development; however, their individual contributions could not be separated with the present experimental design.

### 3.2. Thrust Force and Drilling Behavior

**Figure 10:**
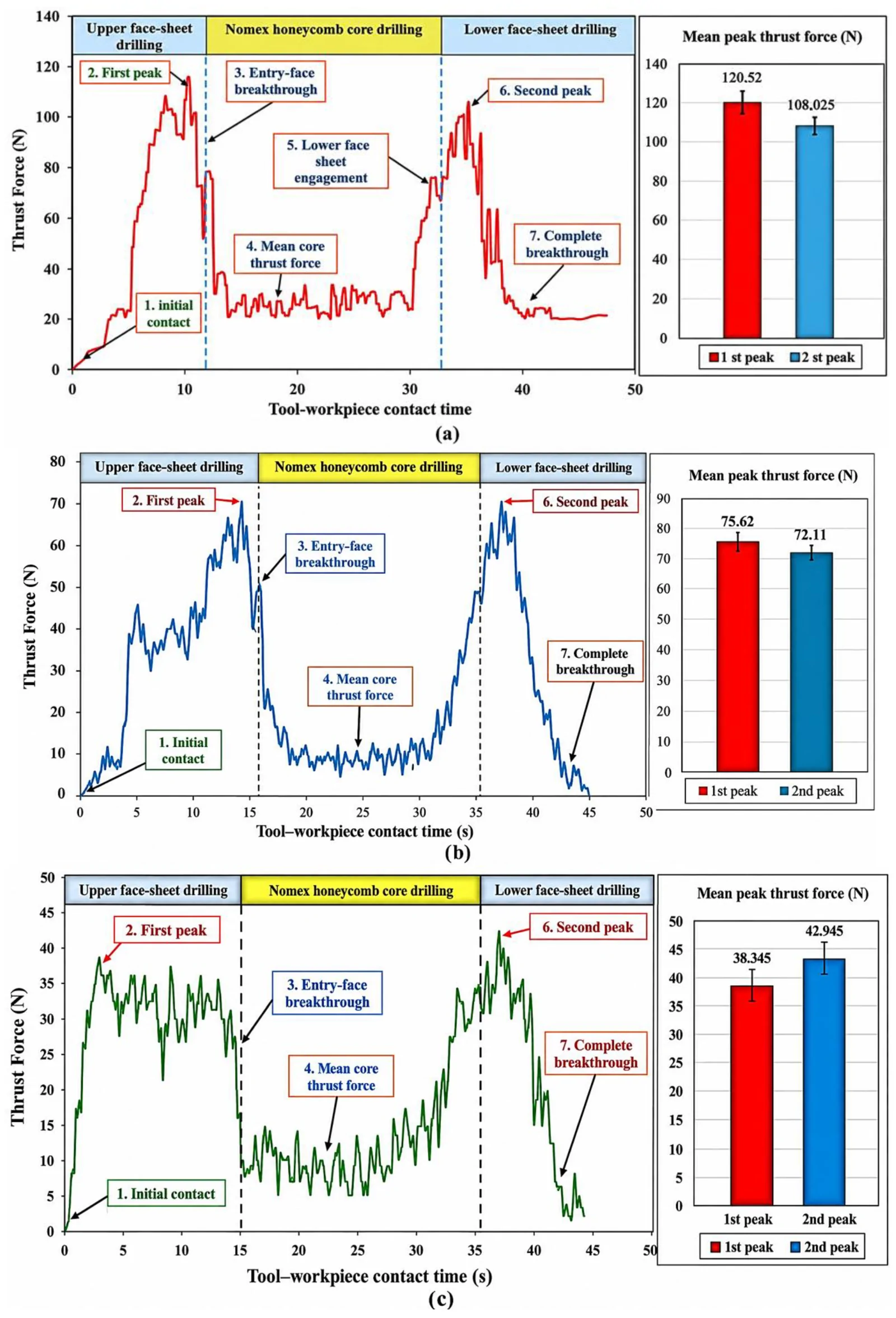

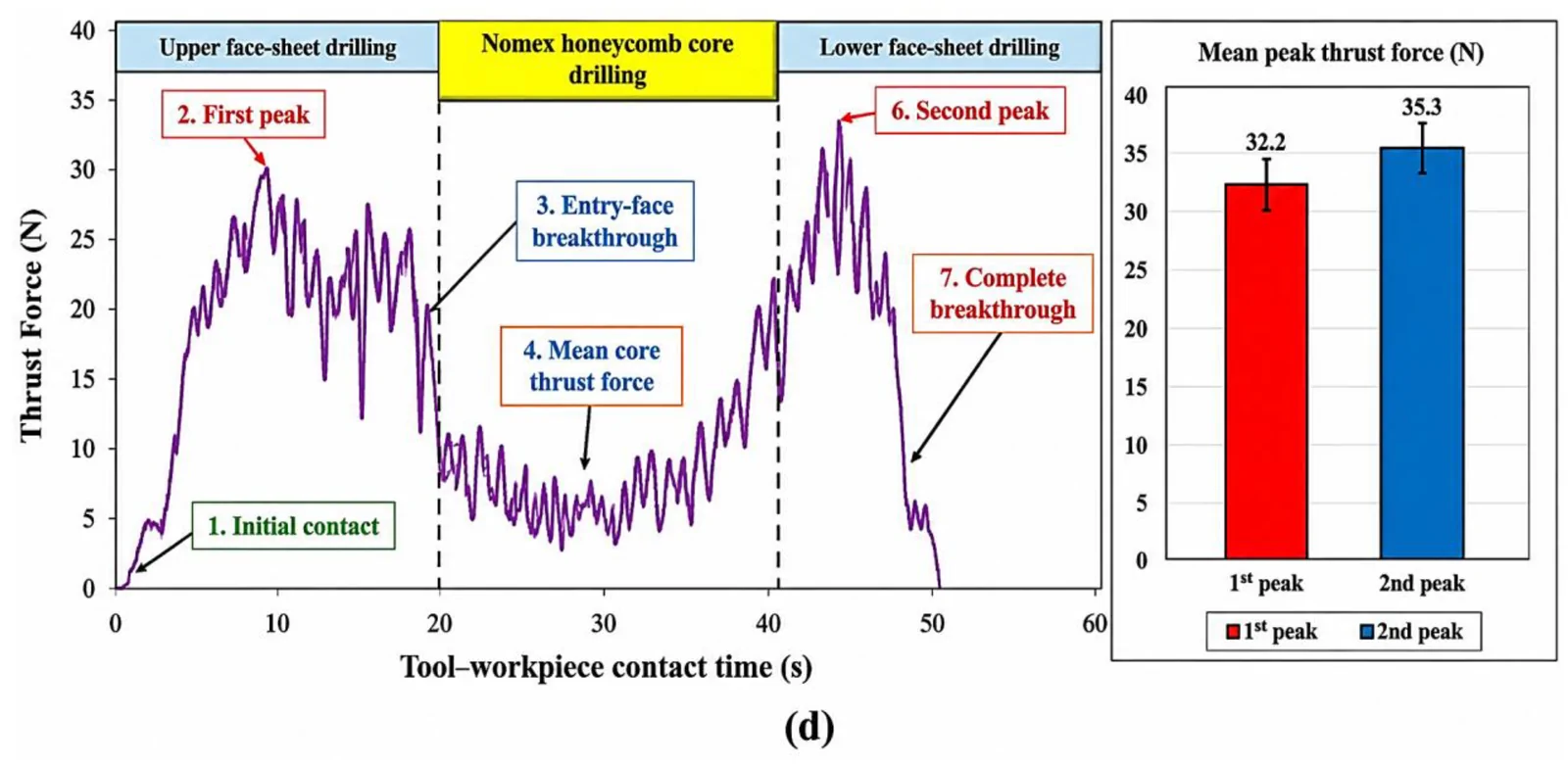
Thrust force–contact time curves, drilling stages, and average peak thrust forces during drilling for different surface layer configurations: (**a**) C/C/C, (**b**) C/G/C, (**c**) G/C/G, and (**d**) G/G/G.

The thrust-time curves and average peak thrust forces obtained during the drilling of C/C/C, C/G/C, G/C/G, and G/G/G sandwich composites are shown in Figure 10. The examination of the curves revealed significant differences in the drilling behavior depending on the carbon/glass ratio in the surface layers. The first force peak occurred during the drilling of the upper surface layer, and the second peak occurred during the drilling of the lower surface layer. The abrupt force drop after the first peak was associated with the transition of the drill from the dense surface layer to the low-density Nomex honeycomb core. The decrease in force after the second peak occurred during the complete drilling of the lower surface layer and after the tool exited the sample. The low and fluctuating force values measured in the core region resulted from the intermittent contact of the drill with the cell voids and walls.

The average peak thrust forces in the C/C/C sandwich composites were 120.52 and 108.03 N, and the highest drilling resistance was obtained in this group. It was evaluated that because of the high elastic modulus and low deformation capacity of carbon fiber, the surface layers bend more limitedly under the drill and create higher resistance to cutting edges. The increase in force over a short time and the formation of high peak values were associated with this rigid structure. In the C/G/C group, peak forces were determined to be approximately 75 and 72 N. The higher deformation capacity of the middle glass layer contributed to a more gradual transfer of the shear loads.

As the glass fiber ratio increased, the abrupt dips and rises in the force curves became more pronounced. The lower rigidity and higher elongation capacity of the glass fiber compared with those of the carbon fiber resulted in more localized bending and elastic recovery in front of the drill. Cutting, pulling, entanglement of fibers around the drill, and irregular fracture of the glass fiber bundles continuously altered the tool-material contact, leading to increased force fluctuations [56].

In the G/C/G and especially the G/G/G curves, lower peak forces and more irregular force variations were obtained compared to the carbon-weighted structures. The rise and fall phases of the thrust force were more undulating in the glass fiber-containing samples. In Figure 10, while the first peak regions of the G/G/G and G/C/G sandwich samples were spread over a wider time interval, the peak was reached, and the force loss after the peak occurred in a shorter transition interval in the C/C/C sample. This difference was attributed to the lower rigidity and higher deformation capacity of the glass fiber, resulting in more local bending, fiber pulling, and elastic recovery in front of the drill bit. However, in the C/C/C sample, the load transfer was more stable owing to the high rigidity of the carbon fiber, and the surface layer penetration time was shorter.

In the G/C/G group, the second peak force was higher than the first peak. This was associated with the lower glass surface layer bending under lower support on the exit side and undergoing greater deformation before shearing. A similar trend was observed in the G/G/G group. Although the all-glass fiber structure had the lowest peak forces, the high fluctuation in the force curves was evaluated in conjunction with more unstable shear, fiber pulling, and exit-side damage.

A strong correlation between the thrust force and delamination has been reported in the literature. Tan and Azmi determined that the onset of delamination depends not only on the applied thrust force but also on the stiffness and critical thrust force of the composite. In the present study, although the highest thrust forces were measured for the C/C/C configuration, the lowest delamination was obtained. This supports the idea that the thrust force alone is not sufficient for evaluating hole quality and that surface layer stiffness and deformation behavior should also be considered [57].

### 3.3. Delamination Damage Analysis

**Figure 11:**
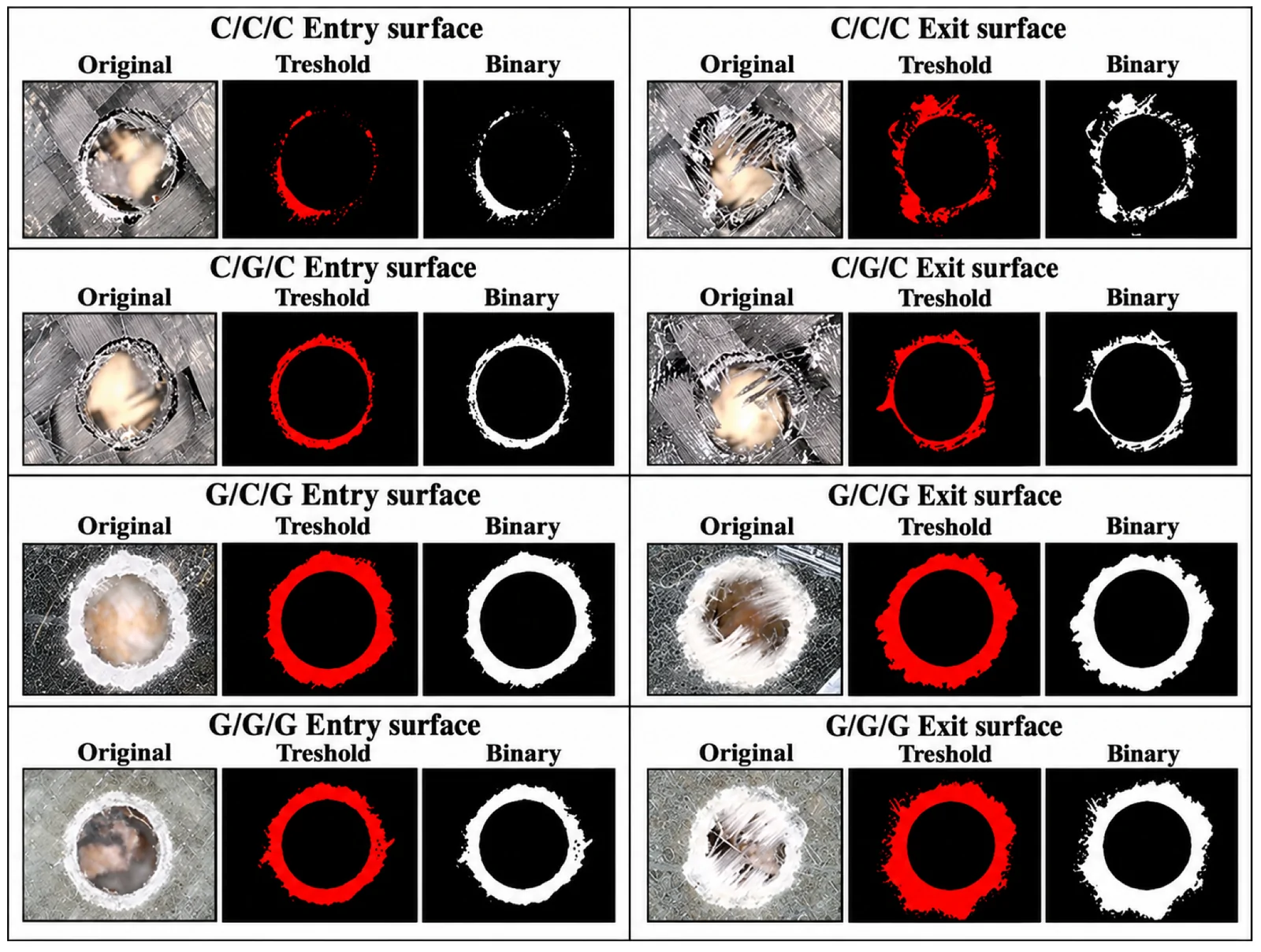
Original, thresholded, and binary images of the drilling-induced damage on the entry and exit surfaces of the C/C/C, C/G/C, G/C/G, and G/G/G configurations. Red regions in the thresholded images and white regions in the binary images represent the identified damaged areas outside the nominal hole boundary.

**Figure 12:**
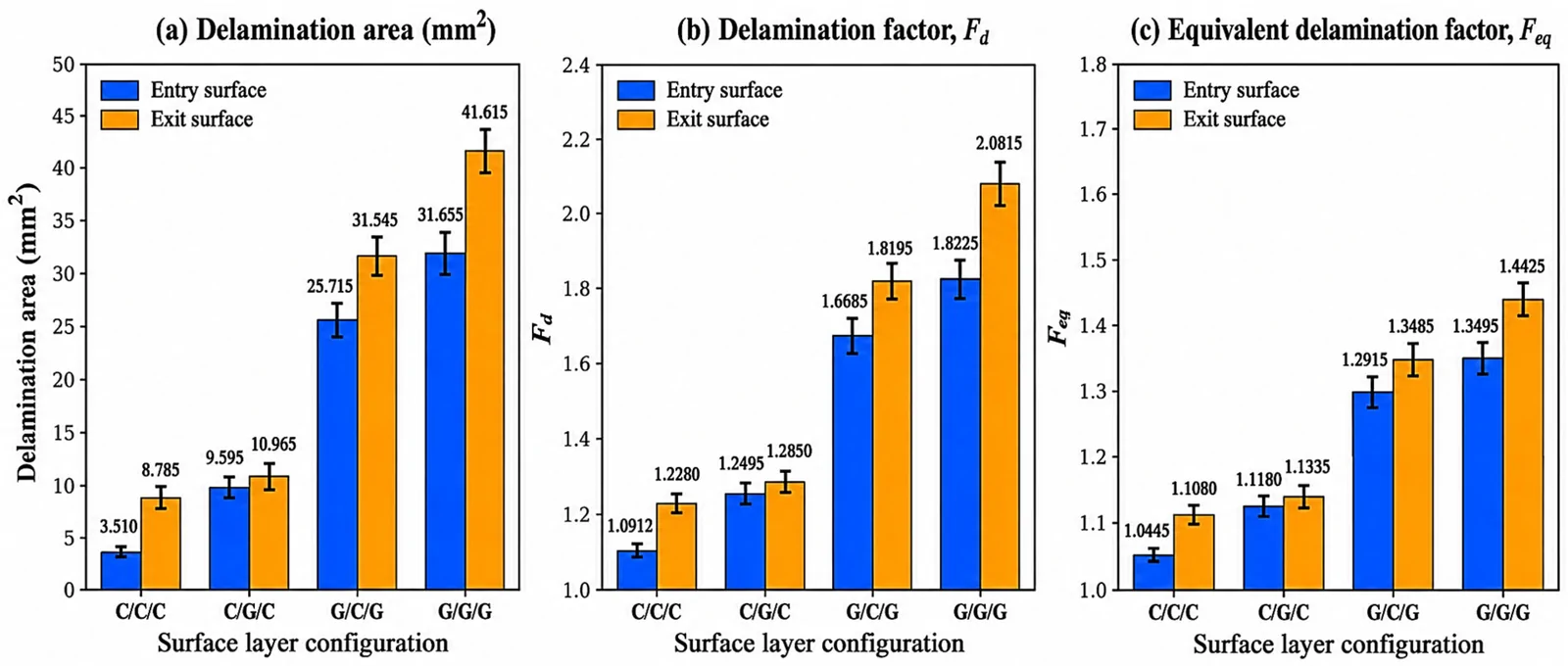
(**a**) Delamination area, (**b**) Delamination factor $F_{d}$ and (**c**) Equivalent Delamination factor $F_{eq}$ at the entry and exit surfaces for different surface layer configurations.

The effect of the surface layer arrangement on hole-induced delamination was evaluated using the original, thresholded, and binary images shown in Figure 11, and the quantitative data in Figure 12 and Table 2. In the binary images, the white regions, and in the thresholded images, the red regions represent the damaged areas outside the nominal hole boundary. In the C/C/C sandwich sample, the damage was narrower and more localized around the hole. As the glass fiber content increased, the damage area expanded, and a more irregular morphology was formed. The largest and most continuous damage areas were observed in the G/G/G-sandwich sample.

The delamination areas at the entry surface were determined to be 3.510, 9.595, 25.715, and 31.655 mm^2^ for the C/C/C, C/G/C, G/C/G, and G/G/G sandwich samples, respectively. At the exit surface, these values increased to 8.785, 10.965, 31.545, and 41.615 mm^2^, respectively. The lowest damage was observed in the C/C/C sandwich sample, whereas the highest damage was observed in the G/G/G sandwich sample. The high rigidity of the carbon fiber layers limited local bending and interlayer separation around the drill hole. The lower rigidity and higher deformation capacity of the glass fiber layers resulted in greater bending and stretching of the fiber bundles under the drilling load. This was associated with a wider separation at the matrix–fiber interface.

A significant difference was observed between C/G/C and G/C/G samples. The entry and exit damage areas of the G/C/G sample were found to be approximately 2.68 and 2.88 times higher, respectively, compared to C/G/C. However, while C/G/C configuration consists of two carbon and one glass layer, G/C/G configuration contains one carbon and two glass layers. The outer layer materials are also different. Therefore, the difference in damage between the two configurations cannot be attributed solely to the outer layer material. It is assessed that the observed difference is due to the combined effect of the carbon/glass ratio and the layer arrangement.

As shown in Figure 12 and Table 2, the damage on the exit surface was higher than that on the entry surface for all sample types. The increase in the exit surface was approximately 150.28%, 14.28%, 22.67%, and 31.46% for the C/C/C, C/G/C, G/C/G, and G/G/G sandwich samples, respectively. Damage on the entry surface is related to the peel-up delamination behavior caused by the drill bits lifting the surface fibers upward. However, on the exit surface, owing to insufficient support under the end layers, the drill’s thrusting force bends the layers outwards, resulting in push-out delamination [56]. Therefore, wider, more continuous, and sometimes fiber-directional damage areas were observed in the exit image. The delamination factors followed the same trend as the measured damage areas. The equivalent delamination factor ($F_{eq}$) increased from 1.0445 for C/C/C to 1.3495 for G/G/G at the entry surface, while the corresponding exit values increased from 1.1080 to 1.4425. A similar trend was obtained for the area-based delamination factor ($F_{d}$). At the entry surface, $F_{d\;}$ ranged from 1.0912 for C/C/C to 1.8225 for G/G/G, whereas the exit values ranged from 1.2280 to 2.0815. The higher $F_{d}$ values reflect the larger affected area around the nominal hole. In particular, the $F_{d}$ value of 2.0815 for the G/G/G exit surface indicates that the total affected area was approximately 2.08 times the nominal hole area.

The standard deviation and CoV values showed relatively low scatter among the repeated measurements. The CoV for the delamination area ranged from 2.51% to 9.02%, while the highest CoV values were 1.66% for $F_{eq}$ and 3.33% for $F_{d}$ These results indicate good repeatability of the measurements.

The sensitivity of the segmentation procedure to the threshold selection was also examined. When the Otsu threshold was varied by ±5% and ±10%, the resulting changes in the damage area were approximately 4.1%, 4.6%, 5.5%, and 7.2% for the G/G/G, G/C/G, C/G/C, and C/C/C configurations, respectively. Considering all configurations, the average variation remained at approximately 5.35%. More importantly, the damage ranking of C/C/C < C/G/C < G/C/G < G/G/G remained unchanged over the investigated threshold range. This indicates that the observed damage trend among the configurations was not sensitive to variations in the selected threshold.

Increasing the carbon fiber ratio, especially that of the outer carbon layers, significantly reduced both the inlet and outlet delaminations. The C/C/C sandwich structure with a full carbon surface layer provided the best hole quality, whereas the G/G/G sandwich structure with full glass fiber exhibited the highest damage. Among the hybrid configurations, C/G/C exhibited lower delamination than G/C/G. Since these configurations differ in both carbon/glass content and ply position, the observed difference reflects their combined influence rather than the independent effect of either parameter.

### 3.4. Ultrasonic C-Scan Analysis of Internal Damage Caused by Drilling

**Figure 13:**
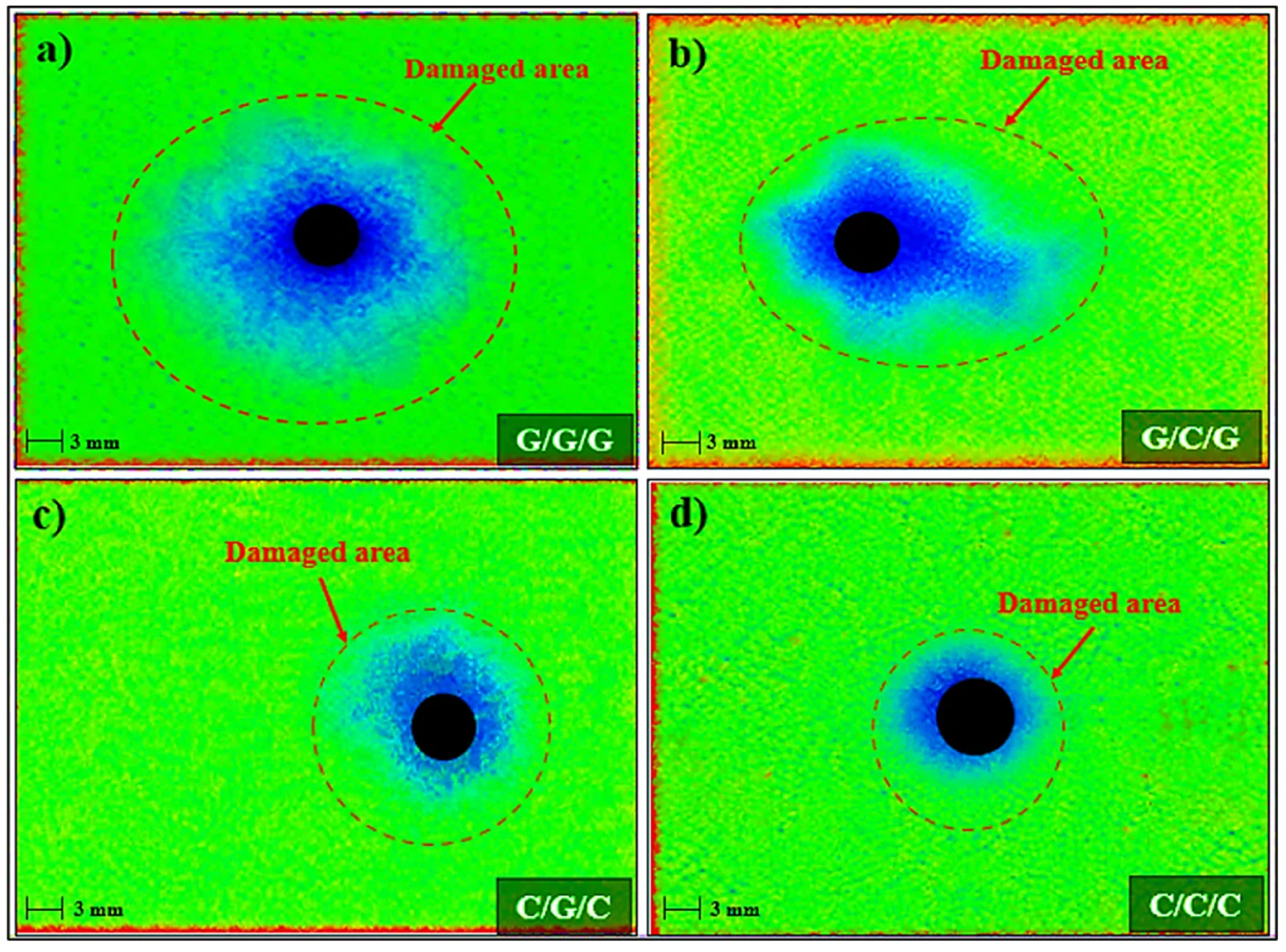
Ultrasonic C-scan images showing drilling-induced internal damage for different face-sheet configurations: (**a**) G/G/G; (**b**) G/C/G; (**c**) C/G/C; and (**d**) C/C/C. The red dashed boundaries indicate the identified internal damaged regions.

The C-scan images given in Figure 13 show that internal damage (BVD) from drilling is significantly affected by the carbon/glass content and layer arrangement of the surface layers. In G/G/G configuration, a broad and approximately circular low-amplitude region formed around the hole, while in the G/C/G sandwich sample, the damage exhibited a more asymmetrical morphology extending in a specific direction. In C/G/C and especially C/C/C configuration, the BVD region remained more limited around the hole.

The high modulus of elasticity of the carbon fiber provided greater resistance to the propagation of internal damage by limiting the out-of-plane bending of the surface layer and deformation transferred to the core-surface interface during drill advancement. This is consistent with the fact that in the C/C/C sandwich specimen, despite the high thrust force, the damage remained localized. As the glass fiber content increased, the higher deformation capacity of the surface layers allowed for greater bending and elastic recovery during drilling. However, fiber–matrix separation, interlayer damage, and separation at the surface layer/core interface were spread over a wider area. The large BVD area observed in the G/G/G sandwich specimen was also due to the low-rigidity glass fiber surfaces transferring the drilling load to a wider area and internal damage propagating further from the hole boundary.

When comparing the hybrid sandwich composites, it was determined that BVD propagation was more limited in the C/G/C sample than in the G/C/G sample. This difference was attributed to the stiffness advantage provided by the higher carbon content combined with outer carbon layers. In the G/C/G structure, the outer glass layers, which allow for more out-of-plane deformation, may have facilitated the propagation of damage towards the core interface and the formation of an asymmetric damage zone. However, because C/G/C and G/C/G configurations differ in both the carbon/glass ratio and layer position, the independent contributions of these two effects cannot be separated using the current experimental design.

In general, C-scan analyses revealed that internal damage from drilling was more limited in carbon-weighted surface layers, whereas BVD regions expanded and acquired a more irregular morphology as the glass fiber ratio increased. Murugan et al. reported that layer arrangement is a determining factor in delamination during drilling of carbon/glass hybrid composites, and that arrangements with carbon fiber positioned in outer layers resulted in lower delamination compared to configurations with outer glass layers. This finding is consistent with the more limited BVD propagation observed in carbon-weighted and outer carbon layer configurations in the present study [58]. Quantitative C-scan results, including mean internal damage area, standard deviation, and coefficient of variation (CoV), are shown in Table 3.

The quantitative evaluation of the C-scan images showed that the internal damage area varied noticeably with the face-sheet configuration. The lowest mean internal damage area was obtained for the C/C/C specimens, with a value of 222.08 ± 17.66 mm^2^. The corresponding values for the C/G/C, G/C/G, and G/G/G configurations were 329.77 ± 19.81 mm^2^, 387.98 ± 19.59 mm^2^, and 469.21 ± 19.88 mm^2^. Thus, the internal damage area followed the order C/C/C < C/G/C < G/C/G < G/G/G. The CoV values remained between 4.24% and 7.95% for all groups, indicating limited scatter between the repeated measurements. The trend obtained from the C-scan measurements was generally consistent with the entry- and exit-surface delamination results determined by optical image processing. Wider internal damage regions were observed as the glass fiber content increased, whereas the C/C/C configuration exhibited the most limited internal damage. These findings indicate that both the face-sheet composition and ply arrangement influence not only the surface damage caused by drilling but also the extent of damage propagation within the sandwich structure.

### 3.5. Dynamic Analysis

**Figure 14:**
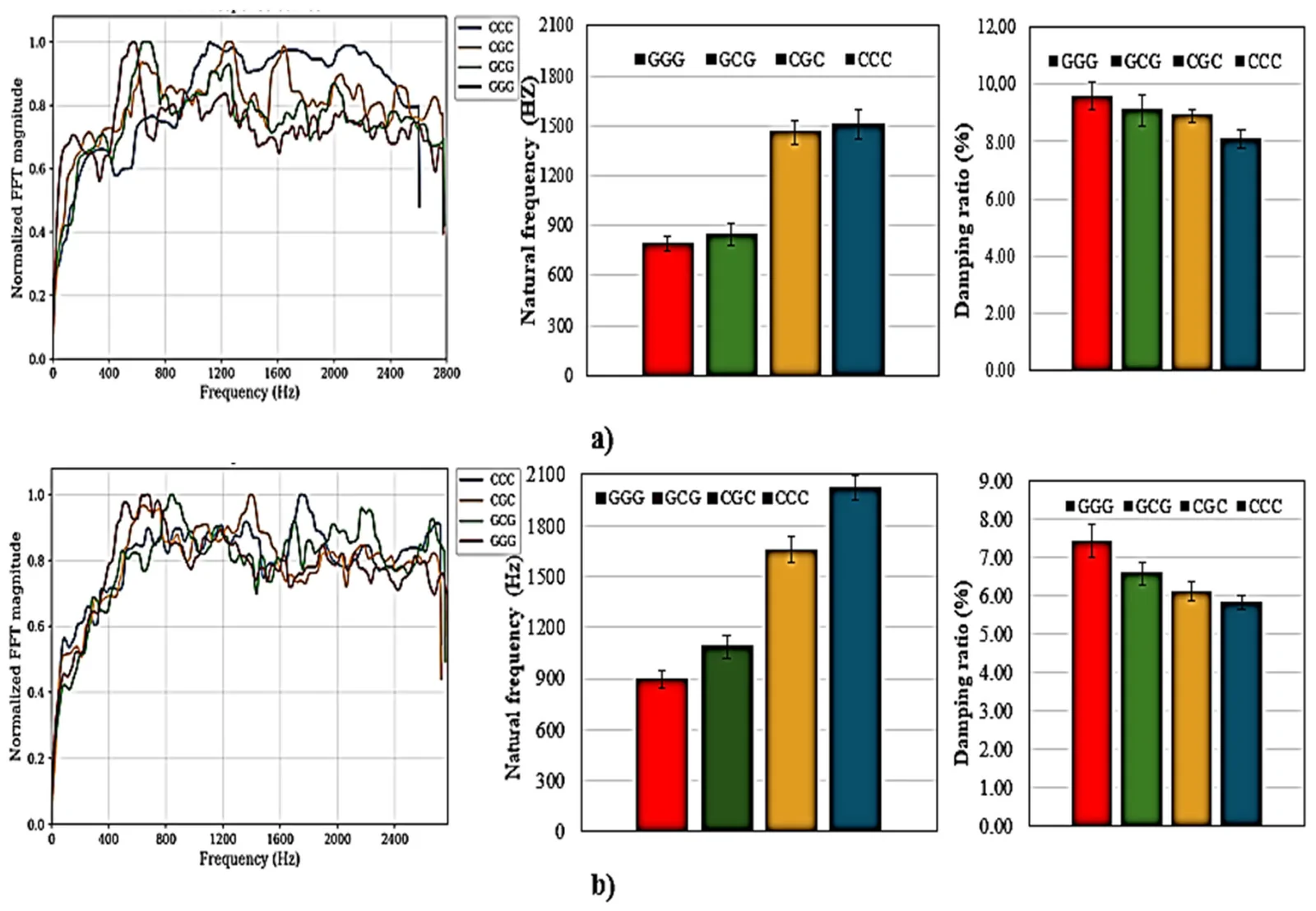
Dynamic characteristics of drilled and undrilled specimens: (**a**) FFT response curves, natural frequency and damping ratio of drilled specimens; (**b**) FFT response curves, natural frequency and damping ratio of undrilled specimens.

The acoustic FFT spectra of the drilled and undrilled specimens were evaluated to identify the dominant resonance peaks. The natural frequencies reported in Figure 14 were estimated from these peaks, while the damping ratios were obtained using the half-power bandwidth method described in Section 2.5. Among the undrilled samples, the highest natural frequency was observed in the C/C/C sandwich sample, whereas the lowest natural frequency was observed in the G/G/G sandwich sample. After drilling, a decrease in the natural frequency was observed for all configurations. The mass reduction caused by drilling was also considered when interpreting this change. The measured mass losses due to drilling were 0.170, 0.227, 0.220, and 0.290 g for the C/C/C, C/G/C, G/C/G, and G/G/G specimens, corresponding to approximately 0.159%, 0.213%, 0.207%, and 0.273% of the initial specimen masses, respectively. Considering the simplified relation $f\propto \sqrt{k/m}$ and assuming unchanged stiffness, these mass reductions alone would theoretically increase the resonance frequency by only approximately 0.080%, 0.107%, 0.103%, and 0.137%, respectively. The experimentally observed decrease in frequency therefore occurred despite the opposing effect of mass removal. This indicates that the reduction in effective structural stiffness associated with drilling-induced damage was more influential than the frequency increase expected from the loss of mass, which is consistent with the established sensitivity of natural frequency to structural stiffness changes [59]. The decrease in natural frequency is particularly pronounced in the C/C/C sandwich sample.

The damping ratio increased in all configurations after drilling. This trend may be related to additional energy dissipation associated with delamination, fiber–matrix interface damage, and friction between damaged surfaces. The post-drilling FFT spectra also showed broader and less distinct resonance regions. Together with the reduction in natural frequency, these changes indicate a clear modification of the dynamic response after drilling and are consistent with the surface and internal damage trends obtained from optical analysis and C-scan. Further studies covering a wider range of damage levels would be useful for developing quantitative relationships between acoustic parameters and measured damage.

Kim and Hwang experimentally and theoretically determined that surface layer/core separation in sandwich beams with carbon/epoxy surface layers and Nomex-aramid honeycomb cores alters bending stiffness and natural frequencies, and that this alteration is reflected in the frequency response functions. In this study, the decrease in the natural frequency identified from the acoustic spectra after drilling was associated with local stiffness loss caused by delamination and interface damage [60]. He et al. showed that the first mode damping ratio increases with increasing delamination rate, and that energy loss due to contact and friction between separated surfaces plays a significant role in this increase. They determined that the modal damping ratio of composite structures increases significantly proportionally with increasing delamination [61].

### 3.6. Quantitative Comparison with Previous Studies

Table 4 presents a quantitative comparison of the results of the current study with those of published studies on carbon/glass hybrid composites.

Han et al. [12] reported increases of 26.54% and 34.38% in the peak bending loads in the L and W directions, respectively, in carbon/glass hybrid surface-layered honeycomb sandwich structures compared with the all-glass fiber reference structure. Monjon et al. [23] determined the bending strengths of all-carbon and all-glass fiber laminates to be 843.3 and 632.5 MPa, respectively. These values showed a difference of approximately 33.33% when the glass fiber structure was referenced. In this study, the average bending load of C/C/C configuration was 36.05% higher than that of G/G/G configuration. These results reveal that the increase in the bending performance obtained with increasing carbon fiber content shows a similar trend to that observed in previous studies. In terms of drilling behavior, Dubey et al. [62] reported that the minimum thrust force for the C4G4 hybrid laminate at 1250 rpm and 50 mm/min was 59.05 N, and the minimum delamination factor at 2000 rpm and 50 mm/min was 1.0001. Although higher thrust forces were measured for C/C/C configuration in the present study, the surface damage remained at the lowest level. The entry and exit damage areas in the C/C/C samples were 88.91% and 78.89% lower, respectively, than those in the G/G/G samples. However, a direct comparison of the drilling results in the literature with absolute values is not appropriate, as the material architecture, number of layers, tool geometry, and drilling conditions differ between studies. Therefore, the comparison is primarily based on the general effects of the carbon/glass layer arrangement on bending and drilling damage.

## 4. Conclusions

The results showed that the carbon/glass face-sheet stacking sequence strongly affected the flexural response, drilling damage, and dynamic behavior of the Nomex honeycomb sandwich composites. The C/C/C configuration reached a mean flexural load 36.05% higher than that of G/G/G and produced the lowest surface and internal damage. Compared with G/G/G, the entry and exit damage areas of C/C/C were reduced by 88.91% and 78.89%, respectively. At the same time, C/C/C generated the highest thrust forces during drilling. This result shows that thrust force alone is not sufficient to describe hole quality. The stiffness, deformation capability, and stacking arrangement of the face sheets also influence the development of drilling-induced damage.

The fact that the C/G/C configuration showed lower surface and internal damage compared to the G/C/G configuration indicates that the carbon/glass composition and layer arrangement combined influence damage development. However, these two configurations differ in both carbon/glass content and ply position. For this reason, the independent contribution of the outer-ply position cannot be separated from the effect of fiber composition in the present experimental design. The ultrasonic C-scan results followed the same general trend as the optical damage measurements, with more limited internal damage in the carbon-rich configurations. Drilling also changed the dynamic response of all specimen groups: the natural frequency decreased, whereas the damping ratio increased after drilling. These acoustic changes were consistent with the surface and internal damage trends obtained from optical analysis and C-scan, indicating the potential of acoustic parameters as damage-sensitive indicators. Further studies with a wider range of damage levels and additional specimens are needed to develop and validate quantitative relationships between acoustic parameters and independently measured damage.

From a design perspective, the face-sheet arrangement should therefore be selected by considering flexural performance, drilling damage, and dynamic response together rather than relying on a single parameter such as thrust force. Future studies should use configurations with a constant carbon/glass ratio while changing only the ply position to separate the effect of stacking more clearly. The influence of different drilling parameters and multiple-hole arrangements also deserves further investigation. SEM examination could provide additional information on local fiber/matrix and interfacial damage, while vibration-based damage indicators could be further assessed together with other non-destructive evaluation techniques.

## Figures and Tables

**Table 1:** Mean flexural loads, standard deviation, coefficient of variation (CoV), and load increment relative to G/G/G sandwich composite for different surface layer configurations.

Configuration	Mean Peak Load ± SD (N)	CoV (%)	Core Shear Stress at Maximum Load ± SD (MPa)	Facing Stress at Maximum Load ± SD (MPa)	Increase Relative to G/G/G (%)
**G/G/G**	1267.49 ± 17.92	1.41	1.32 ± 0.02	23.11 ± 0.33	Reference
**G/C/G**	1390.01 ± 19.25	1.38	1.45 ± 0.02	25.34 ± 0.35	9.67
**C/G/C**	1636.44 ± 13.30	0.81	1.70 ± 0.01	29.83 ± 0.24	29.11
**C/C/C**	1724.42 ± 24.96	1.45	1.80 ± 0.03	31.43 ± 0.46	36.05

**Table 2:** Delamination area, equivalent delamination factor (F_eq_) and area-based delamination factor (F_d_) results or different surface configurations.

Configuration	Surface	Delamination Area Means (mm^2^)	SD (mm^2^)	CoV (%)	(F_eq_) Mean	SD	CoV (%)	(F_d_) Mean	SD	CoV (%)
C/C/C	Entry	3.510	0.110	3.13	1.0445	0.0015	0.14	1.0912	0.0029	0.26
C/C/C	Exit	8.785	0.335	3.81	1.108	0.0039	0.35	1.228	0.0087	0.71
C/G/C	Entry	9.595	0.865	9.02	1.1180	0.0100	0.89	1.2495	0.0225	1.80
C/G/C	Exit	10.965	0.275	2.51	1.1335	0.0035	0.31	1.2850	0.0070	0.54
G/C/G	Entry	25.715	2.135	8.30	1.2915	0.0215	1.66	1.6685	0.0555	3.33
G/C/G	Exit	31.545	2.125	6.74	1.3485	0.0205	1.52	1.8195	0.0555	3.05
G/G/G	Entry	31.655	2.235	7.06	1.3495	0.0215	1.59	1.8225	0.0585	3.21
G/G/G	Exit	41.615	2.365	5.68	1.4425	0.0215	1.49	2.0815	0.0615	2.95

**Table 3:** Quantitative internal damage areas obtained from ultrasonic C-scan analysis.

Configuration	Internal Damage Area (mm^2^)	SD (mm^2^)	CoV (%)
C/C/C	222.08	17.66	7.95
C/G/C	329.77	19.81	6.01
G/C/G	387.98	19.59	5.05
G/G/G	469.21	19.88	4.24

**Table 4:** Quantitative comparison of the present results with selected published studies.

Study	Material/Structure	Evaluated Property	Reported Quantitative Result	Comparison with the Present Study
Han et al. [12], 2025	Carbon/glass hybrid face sheets + aluminum honeycomb core	Flexural behavior	Maximum peak-load increases of 26.54% (L direction) and 34.38% (W direction) relative to the all-glass reference	C/C/C showed a 36.05% higher mean flexural load than G/G/G
Monjon et al. [23], 2022	Carbon/glass epoxy laminates	Flexural strength	8C: 843.3 ± 33.2 MPa; 8G: 632.5 ± 11.8 MPa; calculated difference: 33.33% relative to 8G	The higher flexural performance of the carbon-rich configurations follows the same general trend
Dubey et al. [62], 2024	Carbon/glass hybrid laminate	Drilling behavior	C4G4: minimum thrust force = 59.05 N at 1250 rpm and 50 mm/min; minimum delamination factor = 1.0001 at 2000 rpm and 50 mm/min	C/C/C showed peak thrust forces of 120.52 and 108.03 N while producing the lowest surface damage; direct comparison is limited by differences in material architecture and drilling conditions
Present study	Carbon/glass face sheets + Nomex honeycomb core	Flexural behavior, drilling damage, internal damage, and dynamic response	C/C/C showed a 36.05% higher mean flexural load than G/G/G; entry and exit damage areas were 88.91% and 78.89% lower, respectively	Mechanical response, drilling damage, internal damage, and post-drilling dynamic behavior were evaluated together

## Data Availability

The original contributions presented in this study are included in the article. Further inquiries can be directed to the corresponding author.
